# The prevalence of rotavirus infection among Congolese children younger than 5 years hospitalized for gastroenteritis 10 years after introduction of rotavirus vaccination

**DOI:** 10.1016/j.ijregi.2025.100596

**Published:** 2025-02-13

**Authors:** Cedeche Lebraiche Durain Mboungou, Claujens Chastel Mfoutou Mapanguy, Alain Maxime Mouanga, Vivaldie Mikounou Louya, Jeannhey Christevy Vouvoungui, Raoul Ampa, Francine Ntoumi

**Affiliations:** 1 Fondation Congolaise pour la Recherche Médicale, Brazzaville, Republic of Congo; 2 Faculty of Sciences and Techniques, Marien Ngouabi University, Brazzaville, Republic of Congo; 3 Faculty of Health Sciences, Marien Ngouabi University, Brazzaville, Republic of Congo; 4 Institute of Tropical Medicine, University Hospital Tübingen, Tübingen, Germany

**Keywords:** Gastroenteritis, Prevalence, Rotavirus, Vaccine, Republic of Congo

## Abstract

**Objectives:**

Rotavirus A (RVA) remains the primary cause of acute severe gastroenteritis associated with high rates of childhood hospitalization and mortality worldwide. The prevalence of RVA infection varies within low-income countries. In addition, its genetic diversity may vary among regions and may affect its prevalence. To control the emergence of new strains of RVA circulating in the Republic of Congo, it is essential to know the diversity of both G and P genotypes, which could render the vaccine less effective. Therefore, this study aimed to determine the prevalence and genotype diversity of RVA strains in children who were hospitalized, whether vaccinated or not. Moreover, the potential risk factors of disease severity are identified.

**Methods:**

The study was conducted from April 2022 to March 2023. Stool samples were collected from children younger than 5 years, hospitalized in a pediatric ward for acute gastroenteritis, and a commercial enzyme-linked immunosorbent assay (ELISA) was used to detect RVA cases. In parallel, a multiplex reverse transcription-polymerization chain reaction (RT-PCR) was performed for RVA detection and genotyping. All samples with ambiguous multiplex RT-PCR results were subjected to sequencing.

**Results:**

A total of 227 children hospitalized were enrolled and tested for RVA infection. Using ELISA and PCR, 59 (26%) and 131 (57.7%) individuals were found to be infected with RVA, respectively. All individuals with a positive ELISA test result were confirmed as RVA-positive by PCR. Of the 98 children with documented histories of RVA vaccination, 60% presented with RVA infection. The occurrence of vomiting on more than three occasions within a 24-hour period and the time between vaccination and sample collection were found to be associated with RVA infection. RVA genotyping revealed six P types (P[4]; P[6]; P[8]; P[9]; P[10]; and P[11]). The predominant genotypes were (P[4] 15.3%; P[6] 13.7%; P[8] 22.1%; P[9] 14.5%). Among the eight G types, the most prevalent were G3 (13.7%), G4 (12.2%), and G1 (6.9%). No specific genotype distribution was discernible as a function of vaccination history.

**Conclusions:**

Compared with the findings of our study before the implementation of rotavirus vaccination, in this report, we observed a roughly 20% decreased prevalence of RVA infection in children hospitalized. The results suggest that in Brazzaville at least, 10 years of rotavirus vaccination in the Republic of Congo have had a substantial effect, despite the reported reduced efficacy of the vaccines in low- and middle-income countries. The findings thus support enhanced efforts to increase coverage through national vaccination programs.

## Highlights

• Rotavirus infection in Congolese children is high after vaccine implementation.
• The prevalence of rotavirus A (RVA) was found to be 58% by polymerization chain reaction and 26% by enzyme-linked immunosorbent assay.
• The genetic diversity observed was comparatively high but with low frequencies.
• According to these data, the impact of the RVA vaccine on the prevalence of RVA infection is positive.

## Background

Diarrheal diseases are a leading cause of morbidity and mortality worldwide, and viruses are recognized as the most common etiological agents [1]. Among these, rotavirus A (RVA) remains the primary cause of acute severe gastroenteritis (GE) associated with high childhood hospitalization and mortality worldwide [2]. In sub-Saharan Africa, RVA-associated morbidity and mortality are exceptionally high, with approximately 104,733 children dying annually from the disease [3]. The prevalence of RVA strains varies among countries [4]. Factors such as climate, safe water supply, population density, prevention methods, and healthcare infrastructure influence these variations. Efforts to reduce RVA prevalence in low-income countries include the introduction of RVA vaccines into national immunization programs. In addition, in some low-income countries, vaccination has shown promising results in reducing the incidence and severity of RVA-associated diarrhea [5].

The World Health Organization (WHO) recommended the introduction of RVA vaccines into the national immunization programs of all countries in 2009, particularly in countries with high childhood mortality due to GE [6]. Since then, four rotavirus vaccines (Rotarix™, RotaTeq™, Rotavac®, and ROTASIIL®) have been pre-qualified by the WHO [5]. Two live attenuated oral rotavirus vaccines, a monovalent human vaccine (Rotarix, GlaxoSmithKline Biologicals, Rixensart, Belgium), and a pentavalent bovine-human reassorting vaccine (RotaTeq, Merck Sharp & Dohme LLC, Rahway, NJ, USA) [7], were first licensed and available for use in routine childhood immunization programs worldwide [7]. Rotavac® (Bharat Biotech, India), and Rotasiil® (Serum Institute of India Pvt. Ltd., India) vaccines have also been licensed for use [8]. Rotavirus vaccines have now been introduced and implemented in more than 120 countries, offering an opportunity to assess their effectiveness in diverse settings. These vaccines have been shown to be less effective in areas with more rotavirus-related deaths and lower economic status than in wealthier regions with fewer rotavirus-related deaths [5]. Clinical trials and post-licensure evaluations have shown varying performance, with vaccine efficacy ranging from approximately 70-85% in high- and middle-income countries but from 20-50% in lower-income settings in Africa and Asia [9].

Furthermore, the high prevalence of RVA in low- and middle-income countries may be related to genetic diversity, one of the causative factors of vaccination failure. Owing to the segmented genome, which comprises 11 double-stranded RNA segments, the pattern of RVA genotypes in the human population is evolving through interspecies transmission and/or reassortment events, rendering the vaccine potentially even less effective in the future. On the basis of antigenic and sequence variations of the two outer capsid proteins, VP7 and VP4, rotavirus strains have been categorized into G (glycosylated) and P (protease-sensitive) genotypes, respectively, using a dual classification approach [10]. Genotype diversity may vary among regions and may thus affect the prevalence of RVA. Moreover, the human genotypes that are most commonly recognized are G1P [8], G2P [4], G3P [8], G4P [8], G8P [8], G9P [8], and G12P [5,8]. To control the emergence of RVA strains circulating in any given country, it is therefore crucial to evaluate the genetic variation of both G and P genotypes to predict potential changes in vaccine effectiveness in the future.

Before RVA vaccine introduction in the Republic of Congo, RVA was identified as the predominant etiological agent of diarrhea, with a prevalence of 46.4% in children younger than 5 years living in the southern part of Brazzaville, the capital, and the G1, G2, P [8], and P [6] genotypes were predominant [11]. An RVA vaccine (Rotarix™) was introduced into the Expanded Program of Immunization in the Republic of Congo in April 2014. A study conducted in 2017 in the Republic of Congo [12] showed a reduction of the prevalence of RVA infection in the target population. In 2020, the Rotarix™ vaccine was replaced by the Rotavac® vaccine, one of the monovalent, attenuated oral RVA vaccines. Since then, there have been no published reports on the epidemiological profile of RVA infections in Congolese children younger than 5 years who were hospitalized.

Therefore, the present study aimed to determine the prevalence of RVA infection in children hospitalized. Data are analyzed according to the vaccination and sociodemographic status of the patients.

## Material and methods

### Study design

#### Type, site, and duration of the study

This cross-sectional analytical study was conducted in two health centers in the south of Brazzaville, capital of the Republic of Congo: the referral hospital of Makélékélé and the basic hospital of Bacongo. Samples collected in both locations were transported within 24 hours to the Centre de Recherche sur les Maladies Infectieuses Christophe Mérieux (CeRMI-CM) for further laboratory analysis. The study was conducted from April 2022 to March 2023.

#### Ethical approval

The study protocol was reviewed and approved by the institutional ethics committee of the Congolese Foundation for Medical Research (FCRM) (number 035/CEI/FCRM/2021) and authorized by the Ministry of Health and Population.

#### Patient recruitment and sample collection

After clinical examination and obtaining written informed consent from parents or guardians, children with profiles corresponding to the inclusion criteria were enrolled by the study clinician. The inclusion criteria were as follows: hospitalization for GE, age 5 years or younger, and informed consent of the parents or legal guardian. Clinical definitions for GE were loss of soft stool more than three times within 24 hours with or without abdominal pain, dehydration, vomiting or not, and fever (body temperature ≥37°C).

A liquid stool sample (approximately 3-4 mL) was taken from each child within 48 hours of hospitalization. Sociodemographic data (place of residence, number of people in the house, number of people living with the child, water source, type of toilet, presence of domestic animals, domestic and water waste management and clinical data [dehydration; temperature, times of vomiting and/or loss of soft stool if any], and immunization status [name of the vaccine and number of received doses]), were recorded. Biological samples were transported within 24 hours to CeRMI-CM and stored at −20°C for further analysis.

### Rotavirus antigen detection

Samples were processed and tested using a commercial enzyme-linked immunosorbent assay (ELISA) (ProSpecT Rotavirus Kit, Oxoid, Cambridge, UK). In summary, stool samples were diluted to 10% using the kit diluent. A volume of 100 µl of diluted sample was transferred to the corresponding coated well of the plate (provided in the kit); next, 100 µl of conjugate was added to the well. This operation was performed simultaneously in separate wells with the two controls, with negative and positive test results for RVA, supplied in the kit. The microwells were then incubated for 1 hour at room temperature. After incubation, the microwell contents were aspirated and wells washed five times with diluted wash buffer. To each microwell containing a sample, 100 µl of substrate was added, and the plate was incubated at room temperature for 10 minutes. Finally, to stop the reaction, 100 µl of stop solution was added, and the plate was read within 30 minutes using the spectrophotometer (ELISA plate reader) at 450 nm. The threshold was calculated by adding 0.200 absorbance units to the value of the negative control, in accordance with the manufacturer's instructions.

### Rotavirus RNA extraction

Before processing, fecal samples were diluted 10-fold with phosphate buffered saline (PBS 1X). RNA extraction was performed from the diluted fecal suspension. Briefly, a 10% fecal suspension was prepared by diluting approximately 100 µl of stool solution in 1 mL (if the stool was liquid) or 1 g of stool (if the stool provided was solid). The mixture was centrifuged at 14,000 revolutions per minute for 3 minutes, and 140 µl of the supernatant was used for RNA extraction using the QIAamp Viral RNA Mini Kit (Qiagen, GmbH, Hilden, Germany) according to the manufacturer's instructions. RNA was eluted with 60 µl of elution buffer and stored at −80°C.

### Molecular detection and genotyping by conventional polymerization chain reaction

After extraction of viral RNA, stool samples were tested for the presence of RVA by reverse transcription-polymerization chain reaction (RT-PCR). All samples classified as positive or negative for RVA by immunochromatography were genotyped for VP7 (G-typing) and VP4 (P-typing) genes by the conventional RT-PCR using the designed primers. The first round of RT-PCR involved reverse transcription and amplification of the VP7 and VP4 genes in two separate assays using gene-specific primers. We used the One-Step RT-PCR kit (Qiagen GmbH, 40724 Hilden, Germany) for the rounds of PCR with specific primers targeting the VP7 and VP4 regions. All PCR products were examined by electrophoresis in 100 mL of 1.5% agarose gel stained with SYBR Green [11,13].

### Sequencing of RVA-positive polymerization chain reaction amplicons

All samples with ambiguous multiplex RT-PCR results were subjected to sequencing.

For all samples submitted for sequencing, PCR amplicons were first individually purified using the magnetic bead method, and the eluted DNA was then quantified using Qubit (Qubit DNA BR, Thermo Scientific, Singapore). For Oxford Nanopore sequencing Technology (ONT), each sample was normalized by adjusting the amount of DNA in the tube to a total of 100 ng per sample in a volume of 7.5 µl before adding 2.5 µl of the corresponding barcodes using the SQK-RBK110.96 kit (Oxford Nanopore Technology, Oxford, UK). After incubation (30°C—1 minute; 80°C—1 minute; and 4°C—1 minute) in the thermocycler, all samples (up to 50 per run) were pooled in the same tube and purified by the magnetic bead method using Ampure XP beads (Beckman Coulter, Brea, CA, USA). The pooled eluted DNA (11 µl) constituted the library, and 1 µl of Rapid Sequencing Adapters (Rapid Barcoding, Oxford Nanopore Technology) was immediately added to the library. The mixture was prepared for sequencing load. The cocktail mixture was prepared by adding 37.5 µl sequencing buffer and 25.5 µl loading beads to the library. The prepared mix was used for loading into the ONT GridION flow cell according to the Oxford Nanopore Rapid Barcoding (SQK-RBK110.96) protocol.

### Bioinformatics analysis

**Figure 2:**
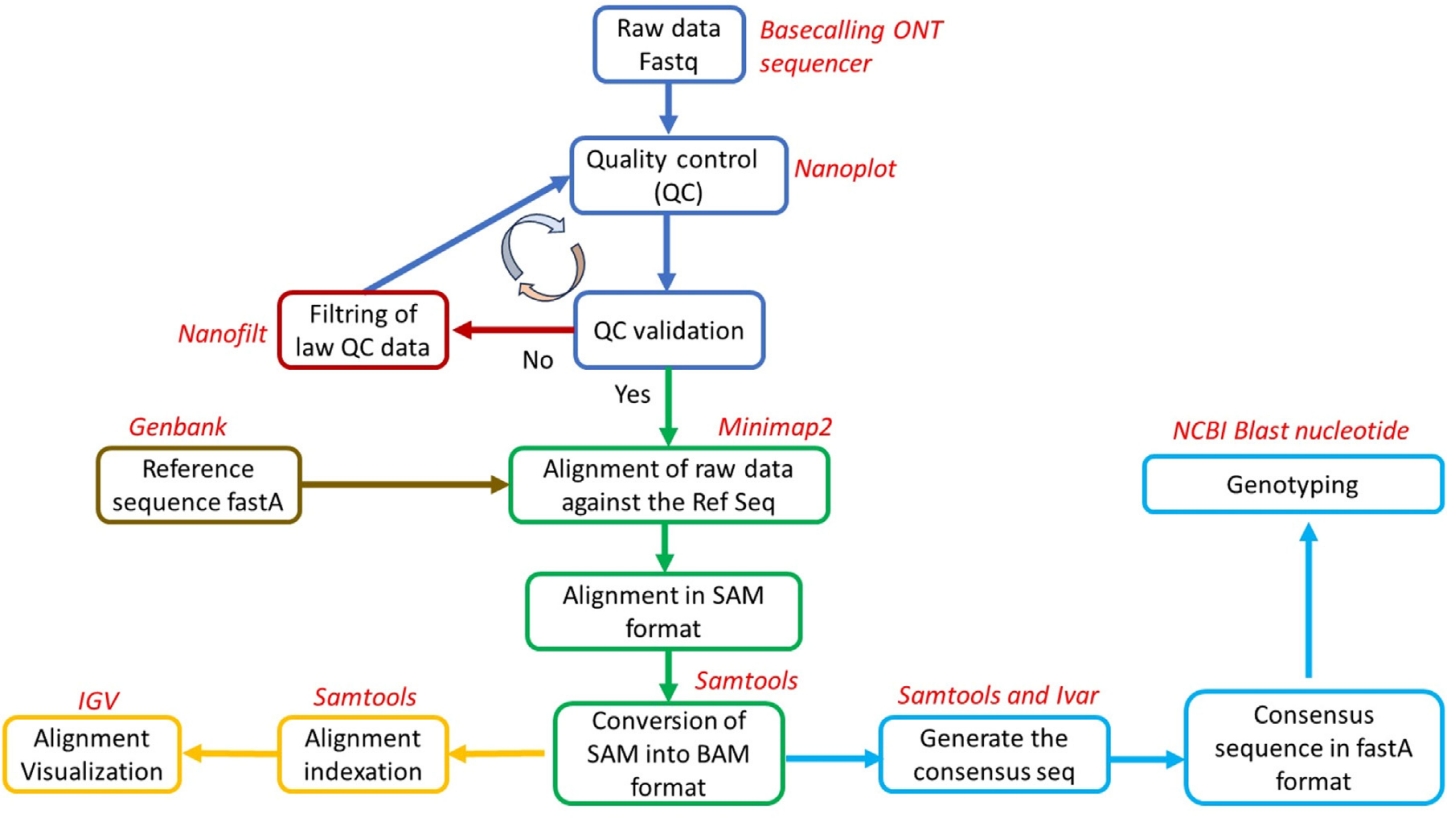
Bioinformatics analysis workflow showing different steps and tools used (in red) at each stage. IGV, Integrative Genomics Viewer; NCBI; ONT, Oxford Nanopore sequencing Technology; SAM.

The FastQ files obtained from sequencing were first compiled in a file corresponding to each barcode used during the library preparation. The corresponding compiled FastQ files underwent the following steps: (i) quality control assessment, (ii) filtering of low-quality reads, (iii) mapping of reads against the reference sequence of the specific human RVA VP7 (X99126.1) or VP4 (KF447864.1) genes retrieved from GenBank, (iv) alignment visualization using the Integrative Genomics Viewer (IGV version 2.16.2) [14,15] to assess the depth and coverage of the alignment, and (v) generation of the final fastA consensus sequence. All these steps were combined into an in-house build pipeline using various software scripts such as Nanoplot (version 1.42.0) [16], Nanofilt (2.8.0) [17], Minimap2 (version 2.28-r1209) [18], Samtools (Version: 1.20) [19], and Ivar (version 1.4.3) [20]. For genotyping, each sequence with more than 80% coverage was submitted individually to the Genbank repository, using the Basic Local Alignment Search Tool nucleotide to obtain the closest genotype corresponding to our sequence. The workflow summarizing the whole process is presented in Figure 2.

### Statistical analysis

Statistical analysis of the data obtained in this study was performed using Graphpad (GraphPad Software ver.9, Boston, MA, USA), including epidemiological (sex, age, vaccination status, season) and clinical variables (number of episodes of diarrhea, vomiting, duration of diarrhea and vomiting, fever, type of dehydration, stool consistency). Chi-squared or Fisher's exact tests were used to compare proportions. Univariate logistic regression was performed to test the effect of each factor on the ELISA or PCR results, and the crude odds ratio was calculated with its 95% confidence interval. Tests were defined as statistically significant if the *P* value was <0.05.

## Results

### Sociodemographic profiles

A total of 227 samples from pediatric inpatients were collected and tested for RVA infection, with a predominance of males (Table 1). Those aged 7-12 months represented the largest group (96 [43.2%], Table 1), whereas overall, 98 children had a record of vaccination with either Rotavac® or Rotarix™. Most of the children presented with fever (axillary temperature ≥ 37.5°C), but other clinical symptoms, such as abdominal pain, were less frequent.

**Table 1:** Rotavirus prevalence by ELISA and by PCR as a function of the patients’ sociodemographic characteristics.

Characteristics	Children with gastroenteritis	Rotavirus prevalence with ELISA detection		Rotavirus prevalence with PCR detection		Odds ratio (with PCR results)	*P*-value
	227	Negatives n (%)	Positives n (%)	Negatives n (%)	Positives n (%)		
		168 (74)	59 **(26)**	96 (42.3)	131 **(57.7)**		
Age							
0-6	54 (23.8)	37 (68.5)	17 (31.5)	21 (38.9)	33 (61.1)	1	
7-12	96 (42.3)	71 (74)	25 (26)	41 (42.7)	55 (57.3)	0.85 (0.43-1.69)	0.73
13-18	45 (19.8)	35 (77.8)	10 (22.2)	22 (48.9)	23 (51.1)	0.66 (0.30-1.48)	0.416
19-24	18 (7.9)	15 (83.3)	3 (16.7)	7 (38.9)	11 (61.1)	1.00 (0.33-2.9)	1
˃24	14 (6.2)	10 (71.4)	4 (28.6)	5 (35.7)	9 (64.3)	1.15 (0.33-3.89)	1
Sex							
Male	134 (59)	102 (76.1)	32 (23.9)	55 (41)	79 (59)	0.88 (0.51-1.51)	0.683
Female	93 (41)	66 (71)	27 (29)	41 (44.1)	52 (55.9)	1	
Place of residence							
North	3 (1.3)	2 (66.7)	1 (33.3)	1 (33.3)	2 (66.7)	1	1.000
Center	34 (15)	27 (79.4)	7 (20.6)	13 (38.2)	21 (61.8)	0.81 (0.07-9.83)	1.000
South	190 (83.7)	139 (80)	51 (20)	82 (43.2)	108 (56.8)	0.66 (0.06-7.39)	
Season							
Wet season	108 (47.6)	96 (88.9)	12 (11.1)	61 (56.5)	47 (43.5)	1	
Dry season	119 (52.4)	72 (60.5)	47 (39.5)	35 (29.4)	84 (70.6)	0.32 (0.19-0.56)	**0.0001**

ELISA, enzyme-linked immunosorbent assay; PCR, polymerization chain reaction

### Detection of rotavirus A infection

**Figure 1:**
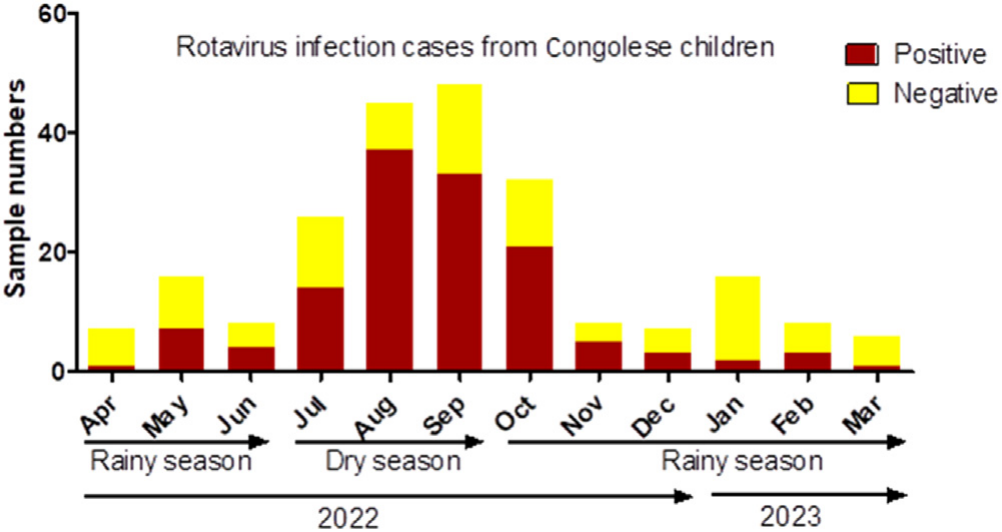
Dynamics of rotavirus cases during the period of sample collection.

A total of 59 individuals had positive test results for RVA by ELISA, and 131 individuals had positive test results by PCR, indicating a prevalence of 26% and 58%, respectively. All 59 samples that had positive test results by ELISA were subsequently confirmed as positive for RVA by PCR. As the figures indicate, the 168 samples deemed RVA-negative by ELISA were subjected to screening by PCR, causing the detection of an additional 72 samples identified as positive for RVA. Sociodemographic and clinical parameters were evaluated in the children enrolled (Table 1). The prevalence was higher among children who vomited at least once within the previous 24 hours. A statistically significant difference was found between these two groups (*P*-value = 0.005). Furthermore, the RVA prevalence was higher during the dry than the rainy season (70.6% vs 42.6%, *P* = 0.0001), with the highest number of cases of RVA infection observed between July and September (Fig. 1). No statistically significant difference in RVA prevalence was observed according to sex.

### Rotavirus A infection and vaccination status

The vaccination status of the children was evaluated in each child's vaccination booklet, showing that 98/227 cases (43%) had histories of RVA vaccination. The vaccine status of 66/227 children (29%) was not known. Of the 98 children vaccinated, 60% were infected with RVA. The prevalence of RVA was slightly higher among the children vaccinated than in the group known to be unvaccinated (52%), but the difference was not statistically significant.

### Risk factors associated with of rotavirus A infection

Among the principal clinical characteristics that may influence the severity of RVA (Table 2), the occurrence of vomiting on more than three occasions within a 24-hour period was associated with RVA infection. Fever (axillary temperature ≥ 37.5°C) was not associated with RVA infection. Other clinical parameters, including the occurrence of diarrhea, the duration of diarrhea, the duration of vomiting, the type of dehydration, the consistency of stools, and the presence of abdominal pain, were not associated with RVA infection. Furthermore, additional parameters, including drinking water sources, the educational level of the children's parents, and the presence of domestic animals in the household, did not indicate any association with RVA infection in these children.

**Table 2:** Prevalence of rotavirus according to the patients’ clinical characteristics and some caregivers’ characteristics.

Characteristics	Children with gastroenteritis n = 227 (%)	Rotavirus detection by polymerization chain reaction		Odds ratio	*P*-value
		Negatives n (%)	Positives n (%)		
Vaccinal status					
Yes	98 (43.2)	39 (39.8)	59 (60.2)	1	0.333
No	63 (27.8)	30 (47.6)	33 (52.4)	1.38 (0.73-2.61)	
Not known	66 (29)	27 (40.9)	39 (59.1)		
Fever					
Yes	170 (75.4)	76 (44.7)	94 (55.3)	1	0.432
No	53 (22.8)	20 (37.7)	33 (62.3)	0.75 (0.40-1.41)	
Not known	4 (1.7)	0	4 (100)		
Diarrheal episode in 24 hours					
≤3	50 (22)	26 (52)	24 (48)	1.66 (0.88-3.12)	0.1434
˃3	172 (75.8)	68 (39.5)	104 (60.5)	1	
Not known	5 (2.2)	3 (60)	2 (40)		
Diarrheal duration (days)					
≤3	59 (26)	27 (45.8)	32 (54.2)	1.28 (0.62-2.68)	0.5765
˃3	58 (25.6)	23 (39.7)	35 (60.3)	1..	.
Not known	110 (48.4)	46 (41.8)	64 (58.2)		
Vomiting episode in 24 hours					
≤3	156 (68.7)	80 (51.3)	76 (48.7)	1	**0.0055**
˃3	67 (29.5)	14 (20.9)	53 (79.1)	2.63 (1.31-5.27)	
Not known	4 (1.8)	2 (50)	2 (50)		
Vomiting duration (days)					
≤3	163 (71.8)	73 (47.8)	90 (55.2)	1.15 (0.52-2.54)	0.8401
˃3	29 (12.8)	14 (48.3)	15 (51.7)	1	
Not known	35 (15.4)	9 (25.7)	26 (74.3)		
Type of dehydration					
Light	110 (48.5)	43 (39.1)	67 (60.9)	1	0.4864
Moderate	100 (44.1)	44 (44)	56 (56)	0.82 (0.47-1.4)	1.000
Severe	6 (2.6)	2 (33.3)	4 (66.7)	1.28 (0.22-7.32)	
Not known	11 (4.8)	7 (63.6)	4 (36.4)		
Stool consistency					
Liquid	154 (67.8)	57 (37)	97 (63)	1	0.0786
Pasty	42 (18.5)	22 (52.4)	20 (47.6)	0.53 (0.27-1.06)	0.1404
Soft	28 (12.3)	15 (53.6)	13 (46.4)	0.51 (0.23-1.15)	
Not known	3 (1.2)	2 (66.7)	1 (33.3)		
Abdominal pain					
Yes	71 (31)	34 (47.9)	37 (52.1)	1	0.245
No	154 (68.1)	60 (38)	94 (62)	0.69 (0.39-1.22)	- -
Not known	2 (0.9)	2 (100)	0	- -	
Drinking water source					
Mineral water	163 (71.8)	65 (39.9)	98 (60.1)	1	0.2873
Faucet	16 (7)	9 (56.2)	7 (43.8)	0.51 (0.18-1.45)	–
Well	1 (04)	0	1 (100)	–	0.3124
River	17 (7.5)	9 (52.9)	8 (47.1)	0.59 (0.21-1.61)	
Not known	30 (13.2)	13 (43.3)	17 (56.7)		
Education level of parents					
Not educated	2 (0.9)	0	2 (100)	–	–
Primary	20 (8.8)	6 (30)	14 (70)	1.17 (0.37-3.69)	1.0000
Secondary	95 (41.9)	46 (48)	49 (52)	0.533	0.1350
High school	60 (26.4)	25 (41)	35 (59)	(0.25-1.14)	0.2967
University	42 (18.5)	14 (31.8)	28 (68.1)	0.600 (0.69-1.38)	
Not known	8 (3.5)	5 (60)	3 (40)	1	
Domestic animal					
Yes	70 (30.8)	33 (47.1)	37 (52.9)	1	0.3836
No	156 (68.7)	63 (40.4)	93 (59.6)	1.32 (0.75-2.36)	–
Not known	1 (0.4)	0	1 (100)	–	

**Figure 3:**
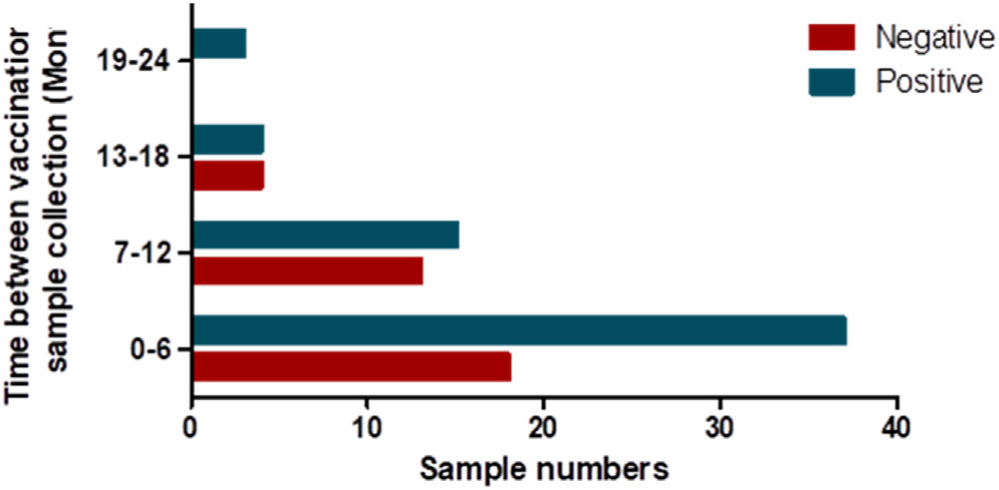
Rotavirus infection frequency after vaccination.

Children whose samples were collected at a time comparatively close to their vaccination date exhibited the highest prevalence of RVA infection (Figure 3). Furthermore, GE was observed predominantly in children having recently received the RVA vaccination, with a decrease in the incidence of GE as a function of the delay between vaccination and sample collection. The highest number of RVA cases was detected in children during the initial 12-month period after the administration of the RVA vaccine, Rotavac™, which was introduced in the country in 2020.

### Genotype distribution

In the 131 RVA infections detected, P genotypes were successfully identified in 102 (Supplementary Table 1). Six different types were found: (P [4]; P [6]; P [8]; P [9]; P [10]; and P [11]), of which four were predominant, namely (P [4] 15.3%; P [6] 13.7%; P [8] 22.1%; P [9] 14.5%), with P [8] being the most prevalent. Most of these P types were identified in samples from children aged between 0 and 18 months, with a lower prevalence in children older than 18 months, and all were present in both vaccinated and unvaccinated children. The single exception was the P [11] type, which was identified in a child older than 24 months.

A total of 72 G types (59 non-identified types) was isolated from the 131 samples that underwent screening. The most prevalent G types were G3 (13.7%), G4 (12.2%), and G1 (6.9%), with the remaining types all present at a prevalence of less than 3%. Most of these RVA G types were identified in children younger than 18 months. Similarly, the same major RVA types G were also identified in children vaccinated. In addition, minor recombinant G types, including G2G8, G3G4G9, and G8G9, were identified in children across all age groups.

Moreover, a total of 54 of the 131 RVA-positive samples (41.2%) were characterized by a combination of the G and P genotypes amongst which 28 distinct genotypes were identified at variable frequencies. Of the 54 samples that were characterized, the G and P genotypes were classified as follows: The most prevalent genotypes were G3P [8] (13%), G2P [4], and G3P [9] (7.4% each), followed by G1P [6], G1P [10], G4P [8], G4P [9], and G4P [11] (3% each) (Supplementary Table I). The remaining genotypes were present at lower frequencies of 2% or less; 77 RVA-positive samples (58.8%) remained unclassified as G or P types. As previously observed, all these genotypes were identified predominantly in children with a minimum age of less than 12 months. The overall genetic diversity was found to be similar in children who were vaccinated and those unvaccinated.

## Discussion

The primary objective of this study was to assess the occurrence of RVA infection in Congolese children hospitalized for GE, with a secondary objective to identify potential risk factors associated with RVA infection.

The prevalence we report here of 58% RVA infections found by PCR was more than twice the 26% identified by ELISA, clearly indicating a notable lack of sensitivity of the stool-based ELISA assay. This is a particular concern with regard to the accuracy of this standard diagnostic test, with worrying implications for clinicians’ treatment choices. Our results are consistent with those published in Egypt [21], with the indication that PCR is probably the most appropriate, accurate method for screening and confirming RVA infection. Indeed, screening RVA infection with ELISA alone may lead to underestimation of the real prevalence of the disease.

In our previous study conducted in 2016 in the same area, the RVA infection prevalence by the same ELISA test was 46% [11]. Given that PCR appears to approximately double the prevalence obtained by ELISA, we can speculate that the true historical prevalence was close to 90%. Regardless, direct comparison of the ELISA data in the present and previous studies does indicate that there has been a decrease of approximately 20% in the prevalence of RVA in children hospitalized with acute GE over the period since the introduction of vaccination in the Republic of Congo. Such a decrease could be explained by improvements in parents’ awareness and education in general hygiene measures, caused in part by the COVID-19 pandemic but also by enhanced community awareness based on knowledge of the high RVA prevalence. Parents’ adherence to vaccination schedules may also have contributed to the observed decrease of RVA prevalence, although, at 43%, the rate of compliance found in this study is not particularly high, as expected. The positive impact of the RVA vaccination campaign is also an important key point to consider. Since 2014, the Congolese immunization program has included a vaccine against rotavirus disease, using the Rotarix™ vaccine. After the introduction of the vaccine, a marked reduction in the ELISA-assessed prevalence of RVA in children hospitalized, from 46% pre-vaccination to 11% in 2018, was observed [12]. It should be noted that in the Republic of Congo, the Rotarix™ vaccine was replaced by the Rotavac® vaccine in 2020. Consequently, the recent study conducted in the Republic of Congo [12] was performed before this change, which may explain the more marked decrease in prevalence those authors observed than that reported here. Thus, the difference is likely attributable to the type of the vaccine, given Rotarix™ contains the G1P8 strain that was predominant in the Republic of Congo before the vaccine was introduced [11]. The Rotavac® vaccine, currently in use, comprises the attenuated RVA G9P11 strain, not a strain that was predominant before its introduction in the country.

The findings presented here indicate that time between vaccination and hospitalization is a critical risk factor for infection. This finding could be because the vaccine strain used in the vaccine currently administered in the Republic of Congo, Rotavac®, is monovalent attenuated human neonatal 116E of G9P11 [22], which can replicate once inoculated in the human body. Although the vaccine strain has been shown to persist in the stool of children vaccinated, the predominant genotypes identified here do not align with that of Rotavac®. Finally, it is notable that the Rotavac® vaccine requires the maintenance of a cold chain at −20°C [22], in contrast to the previously used vaccine, which required storage at +4°C. In such a context, the potentially deleterious effects of the frequent power outages in the Republic of Congo cannot be ignored, given the potential for loss of efficacy of the Rotavac® vaccine due to frequent temperature fluctuations represents a clear and present danger.

The prevalence reported in this study is notably higher than that observed in the Democratic Republic of Congo (34.4%) and in Kenya (14.5%) after the introduction of the Rotasiil® and Rotateq® vaccines, respectively [23]. The prevalence we report here (58%) is similar to that (55%) reported in Gabon [24] before the introduction of the RVA vaccine in Gabon. The discrepancies in the prevalence of RVA infection reported here and those from neighboring countries could be attributed to the sociodemographic variability of the children recruited and to the diagnostic method used to detect infection. In the present study, in addition to the ELISA-based diagnosis, the RT-PCR method was performed on all samples. It is important to note that all ELISA RVA-positive samples were confirmed as positive by RT-PCR. Conversely, a non-negligible number of samples found RVA-negative by ELISA were RVA-positive by RT-PCR, causing an underestimation of the prevalence when using the ELISA method alone for diagnosing RVA infection.

No statistically significant association between RVA infection and various potential risk factors, including drinking water sources, parents’ education level, and the presence of domestic animals in the household, was found. However, this study showed that the prevalence of RVA infection was significantly higher in children with several vomiting episodes. This factor of clinical severity of the disease was already reported by others in Botswana [25], Angola [26], and the Republic of Congo [[11], [12], [13]].

The high genetic diversity observed in this study may constitute evidence of RVA strains evading vaccine-induced immunity in Congolese children. As previously hypothesized [5], the genetic diversity of RVA may potentially compromise the efficacy of any given RVA vaccine, particularly in regions with a high incidence of rotavirus-related mortality and low economic status, such as the Republic of Congo. In addition, vaccination pressure can favor viral strain selection, leading to the emergence of non-vaccine strains. It has also been shown that strains of zoonotic origin can spread among humans with specific genetic traits such as the Lewis phenotype [27]. Although the genetic diversity of RVA reported in other sub-Saharan African countries, such as Uganda, Kenya, Gabon, Angola, and even in previous studies conducted in the Republic of Congo [[11], [12], [13],[27], [28], [29]], differs from that we report here, the results of the present study should be interpreted with caution given the low overall frequency of each strain. It would be beneficial for future research to include additional post-vaccine studies in the Republic of Congo to corroborate the findings of this study.

## Conclusion

The prevalence of RVA was found to be 58% by PCR and 26% by ELISA, the latter being lower than that reported in our own study that pre-dated the introduction of vaccine [11]. The genetic diversity we observed here was comparatively high but with low frequencies of individual strains compared with previous reports in the country. These findings should be considered carefully by healthcare stakeholders because the effectiveness of the RVA vaccine in the Republic of Congo may be affected by genetic diversity, and revised guidance or adapted strategies should be developed by the Expanded National Immunization Program to increase the efficiency of this important public health intervention. At the local level, strategies aimed at improving vaccine coverage of Congolese children should be developed and implemented.

## Declarations of competing interest

The authors have no competing interests to declare.

## Appendix Supplementary materials

Image, application 1

Image, application 2

## References

[1] Mokomane M., Kasvosve I., Melo E.D., Pernica J.M., Goldfarb DM. (2018). The global problem of childhood diarrhoeal diseases: emerging strategies in prevention and management. Ther Adv Infect Dis.

[2] Juliao P., Guzman-Holst A., Gupta V., Velez C., Petrozzi V., Ochoa TJ. (2021). Acute gastroenteritis morbidity and mortality trends following universal rotavirus vaccination in children in Peru: ecological database study with time-trend analysis. Infect Dis Ther.

[3] Manjate F., Quintó L., Chirinda P., Acácio S., Garrine M., Vubil D. (2022). Impact of rotavirus vaccination on diarrheal hospitalizations in children younger than 5 years of age in a rural southern Mozambique. Vaccine.

[4] Nordgren J., Bonkoungou I.J., Nitiema L.W., Sharma S., Ouermi D., Simpore J. (2012). Rotavirus in diarrheal children in rural Burkina Faso: high prevalence of genotype G6P[6]. Infect Genet Evol.

[5] Sadiq A., Khan J. (2023). Rotavirus in developing countries: molecular diversity, epidemiological insights, and strategies for effective vaccination. Front Microbiol.

[6] Tate J.E., Burton A.H., Boschi-Pinto C., Parashar U.D., Organization–Coordinated World Health (2016). Global Rotavirus Surveillance Network, Agocs M, et al. Global, regional, and national estimates of rotavirus mortality in children <5 years of age, 2000–2013. Clin Infect Dis.

[7] Damtie D., Gelaw A., Wondimeneh Y., Aleka Y., Kick M.K., Tigabu Z. (2024). Rotavirus A infection prevalence and spatio-temporal genotype shift among under-five children in Amhara national regional state, Ethiopia: a multi-center cross-sectional study. Vaccines (Basel).

[8] Skansberg A., Sauer M., Tan M., Santosham M., Jennings MC. (2021). Product review of the rotavirus vaccines ROTASIIL, ROTAVAC, and Rotavin-M1. Hum Vaccin Immunother.

[9] Jiang V., Jiang B., Tate J., Parashar U.D., Patel MM. (2010). Performance of rotavirus vaccines in developed and developing countries. Hum Vaccin.

[10] Argüelles M.H., Villegas G.A., Castello A., Abrami A., Ghiringhelli P.D., Semorile L. (2000). VP7 and VP4 genotyping of human group A rotavirus in Buenos Aires, Argentina. J Clin Microbiol.

[11] Mayindou G., Ngokana B., Sidibé A., Moundélé V., Koukouikila-Koussounda F., Christevy Vouvoungui J. (2016). Molecular epidemiology and surveillance of circulating rotavirus and adenovirus in Congolese children with gastroenteritis. J Med Virol.

[12] Léadisaelle H.L., Niama R.F., Mayengue P.I., Leblanc G.G., Cynthia N.B., Igor Judicael L.O. (2023). Molecular study of rotavirus A infection in children with diarrhea, before and after vaccine introduction in Brazzaville and Pointe-Noire, Republic of the Congo. Arch Microbiol Immunol.

[13] Mikounou Louya V., Nguekeng Tsague B., Ntoumi F., Vouvoungui C., Kobawila S.C. (2019). High prevalence of norovirus and rotavirus co-infection in children with acute gastroenteritis hospitalised in Brazzaville, Republic of Congo. Trop Med Int Health.

[14] Robinson J.T., Thorvaldsdóttir H., Turner D., Mesirov JP. (2023). igv.js: an embeddable JavaScript implementation of the Integrative Genomics Viewer (IGV). Bioinformatics.

[15] Thorvaldsdóttir H., Robinson J.T., Mesirov JP. (2013). Integrative Genomics Viewer (IGV): high-performance genomics data visualization and exploration. Brief Bioinform.

[16] De Coster W., Rademakers R. (2023). NanoPack2: population-scale evaluation of long-read sequencing data. Bioinformatics.

[17] De Coster W., D'hert S., Schultz D.T., Cruts M., Van Broeckhoven C. (2018). NanoPack: visualizing and processing long-read sequencing data. Bioinformatics.

[18] Li H. (2018). Minimap2: pairwise alignment for nucleotide sequences. Bioinformatics.

[19] Danecek P., Bonfield J.K., Liddle J., Marshall J., Ohan V., Pollard M.O. (2021). Twelve years of SAMtools and BCFtools. GigaScience.

[20] Grubaugh N.D., Gangavarapu K., Quick J., Matteson N.L., De Jesus J.G., Main B.J. (2019). An amplicon-based sequencing framework for accurately measuring intrahost virus diversity using PrimalSeq and iVar. Genome Biol.

[21] Ibrahim S.B., El-Bialy A.A., Mohammed M.S., El-Sheikh A.O., Elhewala A., Bahgat S. (2015). Detection of Rotavirus in children with acute gastroenteritis in Zagazig University Hospitals in Egypt. Electron Physician.

[22] Varghese T., Kang G., Steele AD. (2022). Understanding rotavirus vaccine efficacy and effectiveness in countries with high child mortality. Vaccines.

[23] Manzemu D.G., Opara J.P., Kasai E.T., Mumbere M., Kampunzu V.M., Likele B.B. (2024). Rotavirus and adenovirus infections in children with acute gastroenteritis after introducing the Rotasiil® vaccine in Kisangani, Democratic Republic of the Congo. PLoS One.

[24] Manouana G.P., Niendorf S., Tomazatos A., Ngwese M.M., Maloum M.N., Moure P.A. (2021). Molecular surveillance and genetic divergence of rotavirus A antigenic epitopes in Gabonese children with acute gastroenteritis. EBiomedicine.

[25] Nitiema L.W., Nordgren J., Ouermi D., Dianou D., Traore A.S., Svensson L. (2011). Burden of rotavirus and other enteropathogens among children with diarrhea in Burkina Faso. Int J Infect Dis.

[26] Gasparinho C., Piedade J., Mirante M.C., Mendes C., Mayer C., Vaz Nery S. (2017). Characterization of rotavirus infection in children with acute gastroenteritis in Bengo Province, Northwestern Angola, prior to vaccine introduction. PLoS One.

[27] Muendo C., Laving A., Kumar R., Osano B., Egondi T., Njuguna P. (2018). Prevalence of rotavirus infection among children with acute diarrhoea after rotavirus vaccine introduction in Kenya, a hospital cross-sectional study. BMC Pediatr.

[28] Mwangi P.N., Potgieter R.L., Uwimana J., Mutesa L., Muganga N., Murenzi D. (2023). The evolution of post-vaccine G8P[4]group a rotavirus strains in Rwanda; notable variance at the neutralization epitope sites. Pathogens.

[29] Manouana G.P., Nguema-Moure P.A., Mbong Ngwese M., Bock C.T., Kremsner P.G., Borrmann S. (2021). Genetic diversity of enteric viruses in children under five years old in Gabon. Viruses.

