# Supplementary material for: The prevalence of rotavirus infection among Congolese children younger than 5 years hospitalized for gastroenteritis 10 years after introduction of rotavirus vaccination
Source: IJID Reg. 2025 Feb 13;14:100596. doi: 10.1016/j.ijregi.2025.100596 (PMC11938071; doi:10.1016/j.ijregi.2025.100596)
Supplement: Supplementary file 1 [file mmc1.docx]

**Supplementary table I:** Genetic diversity according to the vaccinal duration and age groups

| Genotypes | Age groups (months) all positive patients | | | | | Total n(%) |  | Duration (month) between the last vaccine dose and sample collection from 59 positives vaccinated | | | | | Total n(%) |
| --- | --- | --- | --- | --- | --- | --- | --- | --- | --- | --- | --- | --- | --- |
|  | 0 – 6 n(%) | 7 – 12 n(%) | 13 – 18 n(%) | 19 – 24 n(%) | ˃24 n(%) |  |  | 0 – 6 n(%) | 7 – 12 n(%) | 13 – 18 n(%) | 19 – 24 n(%) | ˃24 n(%) |  |
| P(4) | 5 (25) | 9 (45) | 4 (20) | 2 (10) | 0 | 20 (15.3) |  | 1 (11.1) | 7 (77.8) | 0 | 1 (11.1) | 0 | 9 (15.3) |
| P(6) | 5 (27.8) | 9 (50) | 2 (11.1) | 2 (11.1) | 0 | 18 (13.7) |  | 1 (14.3) | 5 (71.4) | 0 | 1 (14.3) | 0 | 7 (11.9) |
| P(8) | 8 (27.6) | 14 (48.3) | 2 (6.9) | 2 (6.9) | 3 (10.3) | 29 (22.1) |  | 4 (30.8) | 7 (53.8) | 2 (15.4) | 0 | 0 | 13 (22) |
| P(9) | 4 (21) | 7 (36.8) | 5 (26.3) | 1 (5.3) | 2 (10.5) | 19 (14.5) |  | 2 (20) | 4 (40) | 4 (40) | 0 | 0 | 10 (16.9) |
| P(10) | 2 (22.2) | 1 (11.1) | 4 (44.4) | 1 (11.1) | 1 (11.1) | 9 (6.9) |  | 1 (25) | 0 | 3 (75) | 0 | 0 | 4 (6.8) |
| P(11) | 2 (28.6) | 3 (42.9) | 1 (14.3) | 0 | 1 (14.3) | 7 (5.3) |  | 2 (40) | 2 (40) | 0 | 0 | 1 (20) | 5 (8.5) |
| undetermined | 7 (24.1) | 12  (41.4) | 5 (17.2) | 3 (10.3) | 2 (6.9) | 29 (22.1) |  | 3 (27.3) | 4 (36.4) | 2 (18.2) | 2 (18.2) | 0 | 11 (18.6) |
| Total | 33 (25.2) | 55 (41.9) | 23 (17.6) | 11 (8.4) | 9 (6.9) | 131 |  | 14 (23.7) | 29 (49.2) | 11 (18.6) | 4 (6.8) | 1 (1.7) | 59 |
|  | | | | | | | | | | | | | |
| G1 | 2 (22.2) | 5 (55.6) | 2 (22.2) | 0 | 0 | 9 (6.9) |  | 0 | 2 (50) | 2 (50) | 0 | 0 | 4 (6.8) |
| G1G9 | 1 (100) | 0 | 0 | 0 | 0 | 1 (0.8) |  | 1 (100) | 0 | 0 | 0 | 0 | 1 (1.7) |
| G1G10 | 0 | 2 (100) | 0 | 0 | 0 | 2 (1.5) |  | 0 | 2 (100) | 0 | 0 | 0 | 2 (3.4) |
| G2 | 0 | 2 (40) | 2 (40) | 0 | 1 (20) | 5 (3.8) |  | 0 | 2 (100) | 0 | 0 | 0 | 2 (3.4) |
| G2G8 | 0 | 0 | 1 (100) | 0 | 0 | 1 (0.8) |  | 0 | 0 | 0 | 0 | 0 | 0 |
| G3 | 3 (16.7) | 9 (50) | 4 (22.2) | 1 (5.6) | 1 (5.6) | 18 (13.7) |  | 0 | 4 (57.1) | 2 (28.6) | 1 (14.3) | 0 | 7 (11.9) |
| G3G4 | 1 (100) | 0 | 0 | 0 | 0 | 1 (0.8) |  | 1 (100) | 0 | 0 | 0 | 0 | 1 (1.7) |
| G3G4G9 | 0 | 0 | 0 | 0 | 1 (100) | 1 (0.8) |  | 0 | 0 | 0 | 0 | 0 | 0 |
| G4 | 7 (43.8) | 7 (43.8) | 1 (6.3) | 0 | 1 (6.3) | 16 (12.2) |  | 4 (66.7) | 1 (16.7) | 0 | 0 | 1 (16.7) | 6 (10.2) |
| G4G9 | 0 | 0 | 1 (50) | 1 (50) | 0 | 2 (1.5) |  | 0 | 0 | 1 (100) | 0 | 0 | 1 (1.7) |
| G4G10 | 1 (100) | 0 | 0 | 0 | 0 | 1 (0.8) |  | 1 (100) | 0 | 0 | 0 | 0 | 1 (1.7) |
| G4G12 | 0 | 1 (100) | 0 | 0 | 0 | 1 (0.8) |  | 0 | 1 (100) | 0 | 0 | 0 | 1 (1.7) |
| G8 | 1 (50) | 0 | 1 (50) | 0 | 0 | 2 (1.5) |  | 0 | 0 | 1 (100) | 0 | 0 | 1 (1.7) |
| G8G9 | 0 | 0 | 1 (100) | 0 | 0 | 1 (0.8) |  | 0 | 0 | 0 | 0 | 0 | 0 |
| G9 | 0 | 1 (25) | 1 (25) | 1 (25) | 1 (25) | 4 (3.1) |  | 0 | 1 (50) | 1 (50) | 0 | 0 | 2 (3.4) |
| G10 | 1 (20) | 4 (80) | 0 | 0 | 0 | 5 (3.8) |  | 1 (100) | 0 | 0 | 0 | 0 | 1 (1.7) |
| G12 | 1 (50) | 1 (50) | 0 | 0 | 0 | 2 (1.5) |  | 1 (50) | 1 (50) | 0 | 0 | 0 | 2 (3.4) |
| Undetermined | 15 (25.4) | 23 (38.9) | 9 (15.3) | 8 (13.6) | 4 (6.8)) | 59 (45) |  | 5 (18.5) | 15 (55.6) | 4 (14.8) | 1 (3.7) | 1 (3.7) | 27 (45.8) |
| Total | 33 (25.2) | 55 (41.9) | 23 (17.6) | 11 (8.4) | 9 (6.9) | 131 |  | 14 (23.7) | 29 (49.2) | 11 (18.6) | 6 (10.2) | 1 (1.7) | 59 |
|  | | | | | | | | | | | | | |
| G1P[4] | 0 | 1 (100) | 0 | 0 | 0 | 1 (0.8) |  | 0 | 1 (100) | 0 | 0 | 0 | 1 (1.4) |
| G1P[6] | 2 (66.7) | 1 (33.3) | 0 | 0 | 0 | 3 (2.3) |  | 0 | 1 (100) | 0 | 0 | 0 | 1 (1.4) |
| G1P[8] | 0 | 1 (100) | 0 | 0 | 0 | 1 (0.8) |  | 0 | 0 | 0 | 0 | 0 | 0 |
| G1G9P[9] | 1 (100) | 0 | 0 | 0 | 0 | 1 (0.8) |  | 1 (100) | 0 | 0 | 0 | 0 | 1 (1.4) |
| G1P[10] | 0 | 1 (33.3) | 2 (66.7) | 0 | 0 | 3 (2.3) |  | 1 (33.3) | 0 | 2 (66.7) | 0 | 0 | 3 (4.2) |
| G2P[4] | 0 | 2 (50) | 2 (50) | 0 | 0 | 4 (3.1) |  | 0 | 2 (100) | 0 | 0 | 0 | 2 (2.8) |
| G2P[8] | 0 | 0 | 0 | 0 | 1 (100) | 1 (0.8) |  | 0 | 0 | 0 | 0 | 0 | 0 |
| G3P[6] | 0 | 1 (50) | 0 | 1 (50) | 0 | 2 (1.5) |  | 0 | 1 (50) | 0 | 1 (50) | 0 | 2 (2.8) |
| G3P[8] | 1 (14.3) | 5 (71.4) | 1 (14.3) | 0 | 0 | 7 (5.3) |  | 0 | 3 (75) | 1 (25) | 0 | 0 | 4 |
| G3P[9] | 1 (25) | 2 (50) | 1 (25) | 0 | 0 | 4 (3.1) |  | 0 | 1 (50) | 1 (50) | 0 | 0 | 2 (2.8) |
| G3P[4] | 1 (50) | 1 (50) | 0 | 0 | 0 | 2 (1.5) |  | 1 (100) | 0 | 0 | 0 | 0 | 1 (1.4) |
| G3P[11] | 0 | 0 | 1 (100) | 0 | 0 | 1 (0.8) |  | 0 | 1(100) | 0 | 0 | 0 | 1 (1.4) |
| G3P[10] | 0 | 0 | 1 (50) | 0 | 1 (50) | 2 (1.5) |  | 0 | 0 | 0 | 0 | 0 | 0 |
| G3G4P[8] | 1 (100) | 0 | 0 | 0 | 0 | 1 (0.8) |  | 1 (100) | 0 | 0 | 0 | 0 | 1 (1.4) |
| G3G4G9P[9] | 0 | 0 | 0 | 0 | 1 (100) | 1 (0.8) |  | 0 | 0 | 0 | 0 | 0 | 0 |
| G4P[4) | 1 (100) | 0 | 0 | 0 | 0 | 1 (0.8) |  | 0 | 0 | 0 | 0 | 0 | 0 |
| G4P[6] | 0 | 1 (50) | 1 (50) | 0 | 0 | 2 (1.5) |  | 0 | 0 | 0 | 0 | 0 | 0 |
| G4P[8] | 2 (66.7) | 1 (33.3) | 0 | 0 | 0 | 3 (2.3) |  | 1 (100) | 0 | 0 | 0 | 0 | 1 (1.4) |
| G4P[9] | 1 (33.3) | 2 (66.7) | 0 | 0 | 0 | 3 (2.3) |  | 1 (50) | 1 (50) | 0 | 0 | 0 | 2 (2.8) |
| G4P[11] | 1 (33.3) | 1 (33.3) | 0 | 0 | 1 (33.3) | 3 (2.3) |  | 1 (33.3) | 1 (33.3) | 0 | 0 | 1 (33.3) | 3 (5.1) |
| G4G10P[11] | 1 (100) | 0 | 0 | 0 | 0 | 1 (0.8) |  | 1 (100) | 0 | 0 | 0 | 0 | 1 (1.4) |
| G4G10P[9] | 0 | 0 | 0 | 0 | 0 | 0 |  | 0 | 0 | 0 | 0 | 0 | 0 |
| G4G12P[9] | 0 | 1 (100) | 0 | 0 | 0 | 1 (0.8) |  | 0 | 1 (100) | 0 | 0 | 0 | 1 (1.4) |
| G4G9P[9] | 0 | 0 | 1 (100) | 0 | 0 | 1 (0.8) |  | 0 | 0 | 1 (100) | 0 | 0 | 1 (1.4) |
| G8P[4] | 1 (100) | 0 | 0 | 0 | 0 | 1 (0.8) |  | 0 | 0 | 0 | 0 | 0 | 0 |
| G8P[8] | 0 | 0 | 1 (100) | 0 | 0 | 1 (0.8) |  | 0 | 0 | 1 (100) | 0 | 0 | 1 (1.4) |
| G8G9P[4] | 0 | 0 | 1 (100) | 0 | 0 | 1 (0.8) |  | 0 | 0 | 0 | 0 | 0 | 0 |
| G9P[9] | 0 | 0 | 1 (50) | 1 (50) | 0 | 2 (1.5) |  | 0 | 0 | 1 (50) | 1 (50) | 0 | 2 (2.8) |
| Partially typed or untyped | 19 (24.7) | 34 (44.2) | 10 (12.9) | 9 (11.7) | 5 (6.5) | 77 (58.8) |  | 8 (20) | 23 (57.5) | 5 (12.5) | 4 (10) | 0 | 40 (56.3) |
| Total | 33 (25.2) | 55 (41.9) | 23 (17.6) | 11 (8.4) | 9 (6.9) | 131 |  | 16 (22.5) | 36 (50.7) | 12 (16.9) | 6 (8.5) | 1 (1.4) | 71 |
