# Supplementary material for: The prevalence of rotavirus infection among Congolese children younger than 5 years hospitalized for gastroenteritis 10 years after introduction of rotavirus vaccination
Source: IJID Reg. 2025 Feb 13;14:100596. doi: 10.1016/j.ijregi.2025.100596 (PMC11938071; doi:10.1016/j.ijregi.2025.100596)
Supplement: Supplementary file 2 [file mmc2.docx]

**Supplementary table II :**

PCR primer list of RVA type G

| **Amorces** | **Génotypes** | **Tailles** | **Séquences (5’-3’)** |
| --- | --- | --- | --- |
| RoA40 | VP7 s |  | ATG TAT GGT ATT GAA TAT ACC AC |
| RoA41 | VP7 as | 882 | AAC TTG CCA TTT TTT CC |
| RoA30 | G1s | 619 | CAA GTA CTC AAA TCA ATG ATG G |
| RoA31 | G2s | 522 | CAA TGA TAT TAA CAC ATT TTC TGT G |
| RoA42 | G3s | 683 | ACG AAC TCA ACA CGA GAG G |
| RoA37 | G4s | 453 | CGT TTC TGG TGA GGA GTT G |
| RoA85 | G8s | 756 | TTR T CG CAC CAT TTG TGA AAT |
| RoA39a | G9s | 176 | CTT GAT GTG ACT AYA AAT AC |
| RoA43 | G10s | 267 | ATG TCA GAC TAC ARA TAC TGG |
| RoA54 | G12s | 388 | CCG ATG GAC GTA ACG TTG TA |

PCR primer list of RVA type P

| **Primers** | **Target genotypes** | **Size of amplicons** | **Sequences (5’-3’)** |
| --- | --- | --- | --- |
| RoA55 | VP4s consensus | 664 | TAT GCT CCA GTN AAT TGG |
| RoA56 | VP4 as |  | ATT GCA TTT CTT TCC ATA ATG |
| RoA44 | P4 as | 353 | CTA TTG TTA GAG GTT AGA GTC |
| RoA34 | P6 as | 137 | TGT TGA TTA GTT GGA TTC AA |
| RoA35a | P8 as | 215 | TCT ACT GGR TTR ACN TGC |
| RoA45 | P9 as | 261 | TGA GAC ATG CAA TTG GAC |
| RoA46 | P10 as | 453 | ATC ATA GTT AGT AGT CGG |
| RoA47 | P11 as | 182 | GTA AAC ATC CAG AAT GTG |
